# No Association Between Vocal Emotion Recognition and Subjective Parental Reporting of Alexithymia in School-Age Children With Hearing Aids

**DOI:** 10.1097/AUD.0000000000001712

**Published:** 2025-09-08

**Authors:** Başak Özkişi Yazgan, Laura Rachman, Gizem Babaoğlu, Pinar Ertürk, Etienne Gaudrain, Leanne Nagels, Stefan Launer, Peter Derleth, Gurjit Singh, Frédérick Uhlemayr, Esra Yücel, Gonca Sennaroğlu, Deniz Başkent

**Affiliations:** 1 Department of Otorhinolaryngology, University Medical Center Groningen (UMCG), University of Groningen, Groningen, the Netherlands; 2 Department of Audiology, Faculty of Health Sciences, Hacettepe University, Ankara, Turkey; 3 Pento Speech and Hearing Centers, Apeldoorn, the Netherlands; 4 Research School of Behavioral and Cognitive Neuroscience, Graduate School of Medical Sciences, University of Groningen, Groningen, the Netherlands; 5 CNRS UMR 5292, Lyon Neuroscience Research Center, Auditory Cognition and Psychoacoustics, Inserm UMRS 1028, Université Claude Bernard Lyon 1, Université de Lyon, Lyon, France; 6 Department of Audiology and Health Innovation, Research and Development, Sonova AG, Stäfa, Switzerland; 7 Sonova Canada, Kitchener, Ontario, Canada; 8 Department of Speech-Language Pathology, University of Toronto, Toronto, Ontario, Canada; 9 Department of Psychology, Toronto Metropolitan University, Toronto, Ontario, Canada.

**Keywords:** Alexithymia, Children, Hearing aids, Vocal emotion recognition

## Abstract

**Objectives:**

Alexithymia is characterized by difficulties in identifying and describing one’s own emotions. Alexithymia has previously been associated with deficits in the processing of emotional information at both behavioral and neurobiological levels, and some studies have shown elevated levels of alexithymic traits in adults with hearing loss. This explorative study investigated alexithymia in young and adolescent school-age children with hearing aids in relation to (1) a sample of age-matched children with normal hearing, (2) age, (3) hearing thresholds, and (4) vocal emotion recognition.

**Design:**

A translated-to-Turkish version of the Children’s Alexithymia Measure (CAM), where higher scores indicate higher levels of alexithymic traits, was filled in by the parents of 37 children (5.5 to 17.8 yr) with bilateral hearing aids and 37 children (5.1 to 18.3 yr) with normal hearing, all native speakers of Turkish. Vocal emotion recognition scores, assessed using the psychophysical vocal emotion recognition test for hearing-impaired populations (EmoHI), were available from a previous study for the group with hearing aids. This test uses non–language-specific pseudospeech sentence recordings expressing three basic emotions: angry, happy, and sad.

**Results:**

Parent-reported CAM scores of children with normal hearing (mean = 7.19 ± 6.61) and children with hearing aids (mean = 8.59 ± 4.38) were within the range previously reported for neurotypical children. Group-level comparison showed no statistically significant difference in CAM scores. However, when considering age, CAM scores of children with normal hearing increased as a function of age, while CAM scores of children with hearing aids were not affected by age. Furthermore, for the youngest children up to 8.8 yr, children with hearing aids had significantly higher CAM scores than children with normal hearing. In children with hearing aids, neither unaided nor aided pure-tone audiometric thresholds were significant predictors for CAM scores. Furthermore, the CAM scores and EmoHI scores were not significantly correlated.

**Conclusions:**

Although the developmental patterns of parent-reported alexithymia scores as a function of age differed between the sample of children with hearing aids and the sample of children with normal hearing, the group difference was observed only for the youngest participants (<8.8 yr). When collapsed across the full age range, there was no group effect, and for both groups, the parent-reported alexithymia scores were within the ranges previously reported in neurotypical children. For the children with hearing aids, unaided and aided hearing thresholds did not have a predictive value for parent-reported alexithymia scores and there was no significant correlation between the CAM and EmoHI scores. These findings together indicate no elevated levels of alexithymic traits in children with hearing aids, at least for 8.8 yr and older. This is not consistent with previous research with adults with hearing loss, assessed with adult-directed questionnaires via self-report. The difference between our findings with children and earlier results from studies with adults with hearing loss may be due to differing methodologies. Alternatively, alexithymia patterns may be different between children and adults with hearing loss. The lack of alexithymia indications implies that the difficulties in vocal emotion recognition for children with hearing aids may be specific to the perception of auditory information and not related to challenges in the general processing of emotional information.

## INTRODUCTION

Emotions shape social interactions in fundamental ways, contributing to our ability to predict and explain the behavior of ourselves and others. The prosodic cues such as voice pitch, intensity, and speaking rate convey the speaker’s affective state (Banse & Scherer 1996). Hearing loss can alter the perception of the prosodic cues, and recognition of vocal emotions could become challenging (Picou et al. 2018). While audibility loss due to hearing impairment can be (partially) restored via a hearing aid (HA), other potential distortions due to hearing loss, such as reduced dynamic range, loudness recruitment, or widened auditory filters (Moore 1996), cannot be easily remedied. Hence, while HAs can greatly help in compensating hearing loss–related reductions and distortions in speech signals, they can only do so to a certain degree, and depending on the extent of physiological changes caused by hearing loss, vocal emotion recognition may still remain challenging even after compensation by HAs (Picou et al. 2018).

In children with hearing loss, vocal emotion recognition difficulties have more often been documented for children with cochlear implants (Chatterjee et al. 2015; Waaramaa et al. 2018), and to a lesser degree with HAs (Most & Aviner 2009; Rachman et al. 2025). A number of factors make it difficult to fully characterize the abilities and limitations of vocal emotion recognition in children with hearing loss. Even in typically developing children with normal hearing (NH), vocal emotion recognition develops differently across individuals, and develops over many years in young children and adolescents (Nagels et al. 2020; Rachman et al. 2025). Because of these developmental effects, it is important to evaluate the emotion recognition abilities of children with HAs in the context of what can be expected of children with NH in the same age range. It is possible that some children with hearing loss could compensate for these difficulties using linguistic content or visual cues (Hopyan-Misakyan et al. 2009). While reduced access to prosodic cues can have an immediate effect on emotion recognition, long-term deprivation or distortion of auditory cues could also have an effect on the development of overall emotion processing. In a previous study (Rachman et al. 2025), to tease apart developmental effects, we compared the vocal emotion recognition abilities of children with HAs to those of age-matched children with NH in a wide age range (5 to 18 yr) for 3 basic emotions (happy, angry, sad). Participants were presented with pseudospeech sentences from the Geneva Multimodal Emotion Portrayals (Bänziger et al. 2012), so that listeners had to rely purely on prosodic cues, with no possible compensation from other modalities. A number of children with HAs showed age-expected recognition scores, indicating potential benefits of HAs for vocal emotion recognition. A subgroup of children with HAs, however, showed lower than age-expected scores. For these children, there were no predictive hearing-related factors, such as aided or unaided hearing thresholds. It was therefore not clear if difficulties in vocal emotion recognition are related to hearing status only, or possible deficits in higher order emotion processing stages.

In this study, we conducted a subjective assessment, an alexithymia questionnaire filled in by parents, in a subgroup of the children with HAs who took part in the study by Rachman et al. (2025). Alexithymia corresponds to an affective condition, associated with difficulty in recognizing and expressing one’s own feelings (Sifneos 1973), and as such, assessments of alexithymia are accepted as measures of self-referential emotion processing recognition (Griffin et al. 2016). In general, a review of studies on emotion processing in adults with varying levels of alexithymic traits (mostly self-reported; only 1 of 12 included studies used an observer rating) has shown that alexithymia is associated with deficits in the processing of emotional stimuli, observed at both behavioral and neurobiological levels, highlighting a link between recognizing and describing one’s own emotions (internal) and identifying the emotions of others (external) (Donges & Suslow 2017). Barrett et al. (2001) have shown that being able to differentiate emotions could be related to higher order stages of emotion processing, such as emotion regulation. In a review of alexithymia studies with adult populations, Grynberg et al. (2012) have shown an association between facial emotion recognition and alexithymic scores based on self-report measures. Several studies have shown an association between self-reported alexithymia and the ability to recognize vocal emotion expressions in adults with Autism Spectrum Disorder (Heaton et al. 2012) and in neurotypical adults with (Goy et al. 2018) and without (Bayot et al. 2014) hearing loss. Elevated levels of alexithymic traits have previously been self-reported in adults with hearing loss (Peñacoba et al. 2020; Blose & Schenkel 2022).

To measure alexithymia, these studies used questionnaires such as 26-Item (Taylor & Bagby 1988) or 20-Item Toronto Alexithymia Scale (TAS-20; Bagby et al. 1994), originally developed in English, or the Bermond-Vorst Alexithymia Questionnaire (Bermond et al. 1994; Vorst & Bermond 2001), originally developed in Dutch. These instruments are all self-report questionnaires developed for adults. Furthermore, the most commonly used questionnaire, TAS-20, has been translated and validated in a large number of other languages. Italian and Finnish translations of the TAS-20 have been used to assess alexithymia in adolescent (≥11 yr) populations as well (Säkkinen et al. 2007; Gatta et al. 2017; Kekkonen et al. 2021); however, it has not been validated for this age group or for younger children. The TAS-20 provides a score between 20 and 100, with scores up to 51 indicating an absence of alexithymia, scores between 52 and 60 indicating possible alexithymia, and scores of 61 and above indicating a presence of alexithymia. Rieffe et al. (2006) have developed an adaptation of the TAS-20, the Alexithymia Questionnaire for Children, and validated this instrument in children from 9.5 to 15.1 years of age. Because of the younger age range of the participants in this study, including children as young as 5 yr old, we used another questionnaire, the Children’s Alexithymia Measure (CAM; Way et al. 2010). The CAM was developed as an instrument with the initial intention to be able to identify alexithymia in children related to post-trauma effects (Way et al. 2010) and neurobiological disorders, such as autism spectrum disorder (Griffin et al. 2016; Trevisan et al. 2016; Scheerer et al. 2021; Moraitopoulou et al. 2024). However, no normative data seem to exist for neurotypical populations, no standardized scores for screening or diagnosis of alexithymia seem to be available, and further, no previous study seems to have used CAM for hearing loss per se. Yet, it is the only instrument usable for young children and adolescents for a wide range of ages (5 to 17 yr). The CAM questionnaire is designed to be filled in by parents or caregivers who know the child well (≥3 mo), enabling assessment of children of younger ages. Since no normative data are available for neurotypical populations, in Table 1, we provide a nonexhaustive overview of example studies where raw CAM scores were reported for neurotypical and neurodiverse child groups. These values provide CAM scores that could be expected for typically developing children.

**TABLE 1.** CAM scores presented from a nonexhaustive list of example studies

Study	Group (n)	Age (yr)	CAM	Group (n)	Age (yr)	CAM
Griffin et al. (2016)	Neurotypical (32)	8–12	5.72 ± 6.24 0–27	ASD or Asperger’s (24)	8–13	19.71 ± 10.21 7–33
Trevisan et al. (2016)	Neurotypical (17)	7–11	8.41 ± 8.88 NA	ASD (17)	7–11	17.29 ± 8.55 NA
Scheerer et al. (2021)	Neurotypical (121)	6–14	7.69 ± 7.52 NA	ASD (120)	6–14	16.71 ± 8.80 NA
Moraitopoulou et al. (2024)	Neurotypical (109)	10–16	11.88 ± 10.23 NA	Autistic traits (75)	10–16	20.67 ± 10.64 NA

The minimum and maximum CAM scores range from 0 to 42, with higher scores indicating increased levels of alexithymia traits. CAM scores are presented as mean ± SD and range (if available).

ASD, Autism Spectrum Disorder; CAM, Children’s Alexithymia Measure; NA, not available.

Children with HAs do not necessarily have trauma experience nor a neurobiological condition per se that could affect emotion processing. Yet, the short- or long-term effects of perceptually-altered auditory affective cues, as was reflected by the vocal emotion recognition EmoHI test (Nagels et al. 2020; Rachman et al. 2025), could potentially also have effects on central mechanisms of emotion processing, and thus, could be reflected in CAM scores of children with hearing loss who make use of HAs. In this exploratory study, we first set out to assess whether children with HAs demonstrate elevated levels of alexithymic traits, measured with the parent-reported CAM questionnaire scores, compared with an age-matched group of children with NH and the extant literature. Second, we aimed to investigate developmental trajectories over age of the CAM scores of children with NH and children with HAs. Third, for children with HAs, we assessed the relationship between CAM scores and both unaided or aided audiometric thresholds. Finally, again for children with HAs, we aimed to assess the correlation between CAM scores and vocal emotion recognition scores, psychophysically assessed with the EmoHI test. Such an association may be indicative of deficits in emotion processing that may occur at higher order processing stages, caused by long-term exposure to perceptually-altered affective acoustic cues and reduced access to auditory information in general, and that are not specific to the ability to perceive relevant acoustic cues at the moment of listening.

## MATERIALS AND METHODS

This study is part of a larger project, Perception of Indexical Cues in Kids and Adults in Turkish (PICKA-tr), including assessments of voice cue discrimination, voice gender categorization, vocal emotion recognition (reported here for a subset of participants), and speech perception with competing speech maskers. The complete project protocol, which lasted close to 1 hr in total, was approved by the Clinical Research Ethical Committee of the University, 2019/07-22 (KA19038). All children and their caregivers (all were parents in this study) were given detailed information about the study, and they provided written informed consent before the study.

### Participants

Parents of 37 school-age children with bilateral HAs (mean age = 10.8 yr, SD = 3.4 yr, range = 5.5 to 17.8 yr) and 37 age-matched children with NH (mean age = 11.5 yr, SD = 3.4 yr, range = 5.1 to 18.3 yr) completed the CAM questionnaire. Participants with HAs had been recruited via the University Audiology Clinic, private HA shops, rehabilitation centers, and word of mouth. Children with NH had been recruited through contacting the hearing siblings or hearing relatives of children with HAs who came to the clinic for routine control appointments. Furthermore, children with NH were also recruited through announcements via social media. For the children with NH who were recruited via the first route, the demographic factors would likely be similar to children with HAs. On the other hand, while there may be some differences in the pool of potential participants with NH and with HAs, the questionnaire data did not show clear demographic differences between the two groups.

All children without a diagnosis of any neurological, developmental, motor, or language disorder had been invited to participate in the study. An additional inclusion criterion for participants with HAs was the use of bilateral HAs for a minimum duration of 6 mo. For both children with HAs and children with NH, details on development or health status were collected through a demographic questionnaire filled in by the parents. Based on this, there were no indications of any neurodevelopmental disorders (e.g., autism spectrum disorder) or major specific speech and language problems (e.g., dyslexia, speech development delay, stuttering, or pronunciation difficulties), other than what could be reasonably expected or directly associated with the participants’ hearing loss. None of the participants had a diagnosis of auditory neuropathy spectrum disorder according to the parent reports nor the medical records of the children with HAs. Parents of the participants reported to spend on average 14 hr per day with their child. Finally, the vocal emotion recognition scores of the children with the HAs were taken from the previously collected data of the PICKA-tr project (reported in Rachman et al. 2025).

**Fig. 1.**
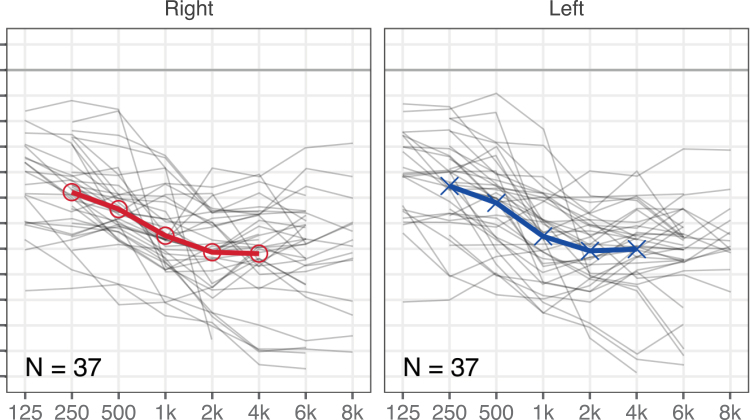
Unaided (left and right panels, for left and right ears) audiometric thresholds, shown for the 37 children with hearing aids. The thin gray lines show the individual hearing thresholds, while the thick blue and red lines indicate the means of the left and right ears, respectively.

In the group with HAs, as a result of the broad inclusion criteria, the degrees of hearing loss varied from moderate to profound, based on the pure-tone average of hearing thresholds (Fig. 1), at four audiometric frequencies between 500 and 4000 Hz (PTA4) (mean PTA4 = 62.4 dB HL, SD = 12.9, range = 40.0 to 97.5). Unaided pure-tone audiometric thresholds were available for all participants with HAs from their medical records. For 25 of the 37 children with HAs, bilateral aided pure-tone audiometric thresholds with daily HA settings were also available from the medical records for the frequencies 250, 500, 1000, 2000, and 4000 Hz, and for some participants, also for 125 (N = 8), 6000 (N = 19), and 8000 Hz (N = 9) (mean-aided PTA4 = 34.7 dB HL, SD = 9.9, range = 18.8 to 50.0). All participants with HAs were users of Phonak HAs, with the models Sky, Naida, Bolero, and Audeo. All HAs were behind-the-ear type, and were fitted with the DSL-V5 prescriptive algorithm, as confirmed by the Phonak target software. For 27 participants, HA data logging was available, and indicated a mean daily HA use of 11.3 hr/d (SD = 3.7, range = 0.4 to 15.8).

### Procedure

All parents and children who were 12 yr or older gave written informed consent at the start of the study. All parents were asked to complete a demographics questionnaire and the CAM questionnaire. Only the children with HAs completed the PICKA-tr psychophysical listening tests, including the EmoHI vocal emotion recognition test.

#### Children’s Alexithymia Measure

For this study, the original CAM was translated to Turkish and back-translated to English by one of the authors (G.B.) and checked by two of the other authors (B.Ö.Y. and P.E.)*. The CAM (Way et al. 2010) is a 14-item parental-report questionnaire, used for the purpose to quantify alexithymic traits in children. All parents received a paper version of the CAM and responded to each question (e.g., “Has difficulty saying he/she feels sad even though he/she looks sad”) on a four-point Likert scale: almost never (0), sometimes (1), often (2), and almost always (3). Each item received a score between 0 and 3, yielding a total score between 0 and 42, with the intention of higher scores indicating increased levels of alexithymia traits. From the 55 children with HAs that had participated in the PICKA-tr study, we received 37 completed CAM forms. Some parents stated as a reason that the surveys were very long after the demographic questionnaire was already filled out. CAM scores were manually entered into a spreadsheet and double-checked by one of the authors (B.Ö.Y.). The CAM typically required 6 to 7 min to complete.

#### Vocal Emotion Recognition Test

The EmoHI test (Nagels et al. 2020) is a vocal emotion recognition test with recordings of two pseudospeech sentences, *Koun se mina lod belam* [kʌun sɘ mina: lɔd be:lam] and *Nekal ibam soud molen* [ne:kal ibam sʌut mo:lɘn], originally taken from the Geneva Multimodal Emotion Portrayals corpus (Bänziger et al. 2012). These sentences were recorded by 2 female and 2 male native Dutch adult speakers conveying three basic emotions (happiness, sadness, and anger). These emotions seem to be learned early in childhood (Widen & Russell 2003), facilitating the EmoHI test applicable for young children. The original recordings also included “neutral,” as the EmoHI test was originally designed for both behavioral and neuroimaging assessments with child and adult participants and “neutral” provides a good baseline for such assessments. However, we found in a pilot study that young children had difficulty understanding the concept of “neutral” in vocal expressions, and for the test interface, the facial expression of “neutral” seemed to be too subtle to make a distinction from the other emotion categories. Therefore, we did not use this category in the current study. Three utterances for each of the 3 emotions and by each of the 4 speakers were presented to participants, resulting in a total of 36 presented experimental stimuli. Four utterances that were not used during the test phase were presented as practice trials before the experiment. The test was conducted using a touchscreen laptop with a child-friendly interface showing a cartoon presentation of a circus setting (Nagels et al. 2020). In each trial, participants heard the stimulus once and were asked to select one of the three clowns whose facial expression corresponded to the perceived vocal emotion by tapping on the screen. The duration of the EmoHI test was approximately 5 to 7 min.

### Data Analysis

We calculated the mean, SD, and range of the CAM scores, and of the EmoHI scores for each emotion, as well as averaged across the three emotions, per group. In further statistical analyses, we compared the CAM scores of children with HAs and children with NH and assessed whether the scores change as a function of age for each group. For the children with HAs, we also examined whether their overall EmoHI scores develop as a function of age and assessed the correlation between CAM scores and EmoHI scores. All statistical analyses were performed using R (version 4.2.3).

First, we calculated the CAM scores for each participant, ranging between 0 and 42. For each group, the mean score, SD, and range are presented. We applied a generalized additive model (GAM) to compare CAM scores between children with HAs and children with NH and examine the effect of age, using the mgcv (v.1.8.42), itsadug (v.2.4.1), and gratia (v.0.8.1) packages in R. GAMs enable modeling both linear and nonlinear relationships between dependent and independent variables, making them appropriate for the assessment of developmental effects. To examine the effect of age on CAM scores for each group, we used the following model:


$$\begin{array}{l} CAMscore∼group+s(age,by=group) \end{array}$$

We used a cubic regression spline with shrinkage for the age factor, fitted per group (NH, HA), with the *k* parameter set to 10. We compared the groups by estimating the spline of the difference between the groups, using the 95% confidence interval of the difference spline to define the age range where the groups significantly differed. The ages where the confidence interval of the difference spline did not cross 0 were considered significantly different. In addition, for children with HAs, we explored whether unaided or aided hearing thresholds had an effect on the CAM scores. For this analysis, we compared the GAM fits to the CAM scores of the children with HAs, with unaided (for all children with HAs, N = 37) or aided (N = 25) PTA4 added as a predictor. Note that the first GAM, comparing children with NH and children with HAs, was fitted using the restricted maximum likelihood method because it is regarded as unbiased (Snijders & Bosker, 2011). The GAMs assessing the predictive value of unaided or aided PTA4, however, were fitted using the maximum likelihood model, as recommended by (Faraway 2016) and Snijders and Bosker (2011) for the comparison of models that differ by their fixed effects.

Second, we calculated the EmoHI test scores of the children with HAs in percentage correct, for each emotion as well as combined across the three emotions, and reported the mean score, SD, and range. To examine the effect of age on overall EmoHI scores, we used another GAM as follows:


$$\begin{array}{l} \text{c}orrect∼\;\;\;s(age)+s(\;participant) \end{array}$$

The dependent variable “correct” took a value of 0 or 1 and the model was generalized to a binomial distribution with a logit link function. We used a cubic regression spline with shrinkage for the age factor. A random effect for “participant” was included to account for the twelve repetitions of each condition (12 trials per emotion category) within the same participant. The other model parameters were the same as in the first GAM analysis.

Finally, we assessed the relation between CAM scores and overall EmoHI scores with a Pearson’s correlation.

## RESULTS

### Descriptive Results of Parent-Reported Alexithymic Traits and Vocal Emotion Recognition

For the alexithymia questionnaire, the children with HAs had a mean CAM score of 8.59 (SD = 4.38, range = 2 to 19), and the children with NH had a mean CAM score of 7.19 (SD = 6.61, range = 0 to 29). For vocal emotion recognition, previous data (Rachman et al. 2025) had shown a significant overall group difference in EmoHI scores between a larger group of 55 children with HAs and 86 children with NH (Table 2). For the subgroup of 37 children with HAs whose data are used in this study, children with HAs had an overall mean EmoHI score of 49.47% (Table 2).

**TABLE 2.** EmoHI scores in %-correct of the children with NH and with HAs from Rachman et al. (2025), as well as from a subgroup of 37 children with HAs considered in the current study

	Children With NH (N = 86, Rachman et al. 2025)	Children With HAs (N = 55, Rachman et al. 2025)	Children With HAs (N = 37, Current Study)
Overall EmoHI score			
M (SD)	61.37 (14.21)	48.18 (12.82)	49.47 (13.11)
Range	33.33–91.76	25.00–80.56	25.00–80.56
Angry score			
M (SD)	59.59 (14.66)	45.15 (14.76)	48.42 (13.44)
Range	33.33–100.00	16.67–83.33	25.00–83.33
Happy score			
M (SD)	56.20 (18.04)	44.24 (17.11)	43.02 (18.37)
Range	25.00–100.00	8.33–75.00	8.33–75.00
Sad score			
M (SD)	68.31 (24.52)	55.15 (20.19)	56.98 (20.74)
Range	25.00–100.00	8.33–100.00	8.33–100.00

HAs, hearing aids; M, mean; NH, normal hearing.

### Group Comparison for Parent-Reported Alexithymic Traits

**Fig. 2.**
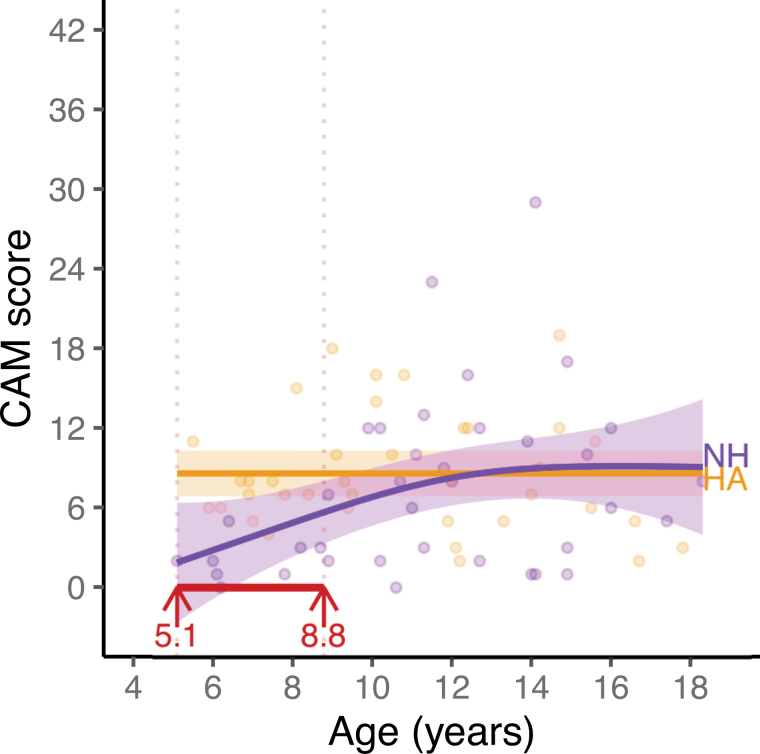
Relationship between CAM scores and age for children with HAs and children with NH. The black lines represent the correlations between CAM scores and age and between EmoHI scores and age. The dashed blue lines indicate the minimum and maximum scores for CAM and EmoHI. The dashed red line indicates chance level performance for the EmoHI test (33.3%). The shaded areas represent the 95% confidence interval. CAM indicates Children’s Alexithymia Measure; HAs, hearing aids; NH, normal hearing.

The results of the first GAM analysis, comparing CAM scores of the two groups, and assessing the CAM scores as a function of age, are presented in Figure 2. This analysis showed that the CAM scores did not differ significantly at the group level [*χ*^2^(1.00) = 1.80, *p* = 0.18]. When considering the effect of age, the GAM results revealed that the CAM scores of the children with HAs were significantly higher than the CAM scores of the children with NH in the age range between 5.1 (youngest age tested) and 8.8 yr. There was no significant group difference anymore for children older than 8.8 years. For developmental trajectories, for the children with NH, the GAM results showed a significant effect of age on the CAM scores [*F*(1.519, 70.481) = 0.82; *p* < 0.01], which seems to plateau around 12 to 14 yr based on visual inspection of the GAM estimation (Fig. 2). For the children with HAs, there was no significant age effect on the CAM scores [*F*(0.000, 70.481) = 0.00; *p* = 0.63].

### Potential Effects of Hearing-Related Factors and Vocal Emotion Recognition on Parent-Reported Alexithymic Traits

**Fig. 3.**
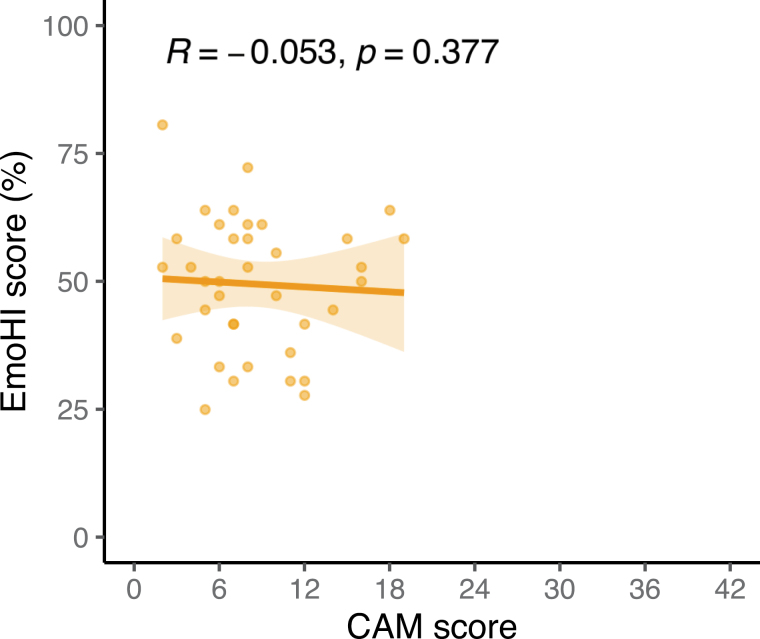
Relationship between EmoHI and CAM scores shown for the children with HAs. The solid line represents the correlation between EmoHI scores and CAM scores. The shaded area represents the 95% confidence interval. CAM indicates Children’s Alexithymia Measure; HAs, hearing aids.

In a second exploratory GAM analysis, considering only the group of children with HAs, the predictive value of unaided and aided PTA4 for the CAM scores was assessed by comparing GAM fits where the PTA4 was added as a predictor. This analysis showed that neither unaided [ΔAIC = −1.74, *χ*^2^(1.00) = 0.13, *p* = 0.61] nor aided [ΔAIC = −1.14, *χ*^2^(1.00) = 0.43, *p* = 0.36] PTA4 had significant predictive value for CAM scores of children with HAs. The third GAM analysis did not show a significant effect of age on the EmoHI scores of children with HAs [*χ*^2^(0.035) = 0.04, *p* = 0.33]. The correlation analyses indicated no significant correlation between CAM and EmoHI scores for children with HAs (one-tailed test for negative correlation: *r* = −0.053, *p* = 0.38; Fig. 3).

## DISCUSSION

The purpose of this study was to explore the presence of alexithymic traits in a population of children with hearing loss who use bilateral HAs, compared with age-matched children with NH, and also in relation to age and unaided and aided hearing thresholds. Furthermore, for children with HAs, we assessed whether the level of alexithymic traits, as measured by the subjective parent-report questionnaire CAM, correlated with scores of a psychophysical vocal emotion recognition test, EmoHI.

### Developmental Effects on Alexithymic Traits

To the best of our knowledge, this study is the first to investigate age effects on parent-reported alexithymia, measured with the CAM, for a wide range of ages in school-age children (5 to 18 yr). We found that the CAM scores of children with NH were significantly dependent on age, with older children having higher CAM scores. For children with HAs, the CAM scores did not change as a function of age. In addition, for the younger children up to 8.8 yr, CAM scores differed significantly between the two groups, with higher CAM scores for the HA group, while this difference disappeared for children older than 8.8 yr. While CAM scores showed an increase in younger children with NH, they seemed to reach a plateau around age 12 to 14. The stability of CAM scores in teenagers with NH is in line with Moraitopoulou et al. (2024) who collected parent-reported CAM scores for adolescents (10 to 16 yr) and found no significant age effect.

Our results deviate from some other studies that used the self-reported TAS-20 to assess alexithymia and reported decreasing scores (i.e., lower levels of alexithymic traits) as a function of age based on large samples (N > 750) of adolescents and young adults (Säkkinen et al. 2007; Gatta et al. 2017; Kekkonen et al. 2021). However, while Gatta et al. (2017) found an overall decline in TAS-20 scores as a function of age in their sample as a whole (11 to 17 yr), they also report an increase in TAS-20 scores for their youngest participants up to age 14. It is possible that the manifestation of alexithymia during childhood follows a u-shaped trajectory. U-shaped functions have been identified in various developmental processes in childhood (e.g., in the development of motor or language skills; for a review, see Pauls et al. 2013). Due to the limited number of studies investigating an age effect on alexithymia in younger children (<12 yr), whether with the self-reported TAS-20 or with the parent-reported CAM, and the limited sample size of our participant groups in relation to the wide age range considered, we cannot draw strong conclusions on how the developmental trajectory in this study relates to what can be expected in children with NH.

Some caution is also warranted when comparing these studies due to methodological differences, such as self-reported versus parent-reported measures. Some of the previous studies have shown weak correlations between self-reported and parent-reported measures of alexithymia and it has been suggested that parents may not be able to accurately report on the presence or level of alexithymic traits their children may have, or alternatively, self-reports and parent reports may capture different aspects of the alexithymia construct (Griffin et al. 2016; Hobson & van der Bedem 2021; Lampi et al. 2021). The age effect on CAM scores found in children with NH could therefore be a result of an age-related change in alexithymia traits, or, alternatively, a change in the extent to which parents are able to evaluate the presence or level of alexithymia traits at different childhood stages, or a combination of both. In addition, it is possible that parents adjust their expectations for what is an appropriate emotional or otherwise communicative expression for their child’s age when completing the questionnaire, such that higher expectations for older children and adolescents may lead to higher CAM scores, indicating higher levels of alexithymic traits, with respect to younger children. This would however not explain the higher CAM scores for the younger children with HAs compared with children with NH, and further research in larger cohorts is needed to map the developmental trajectories of alexithymia scores in children with NH and in children with HAs for a more comprehensive understanding of developmental aspects.

While the overall group comparison did not reveal a significant difference in CAM scores between children with NH and children with HAs, the parents of the younger children with HAs reported significantly higher CAM scores compared with the parents of the age-matched children with NH. It is possible that the younger children with HAs in this study have more difficulties describing their own emotions compared with the control group of children with NH, a difference that may disappear as the children become older. However, even though there was a significant group difference in the younger age range, the parent-reported CAM scores of both the children with HAs and the children with NH in this study were within the ranges reported in earlier studies for typically developing children (Griffin et al. 2016; Moraitopoulou et al. 2024; Scheerer et al. 2021; Trevisan et al. 2016; summarized in Table 1). This suggests that for both groups in this study, the CAM scores did not provide any indication for pathological levels of alexithymic traits, based on previous literature. Finally, it should be noted that the previously mentioned studies used the original English version of the CAM, while the current study made use of a Turkish translation that was not officially validated. Any comparison between studies should thus be done with caution as cultural or language differences may have an impact on how the questionnaire was translated and how questions are interpreted by parents.

### Hearing-Related Factors

To further examine potential effects of hearing loss on parent-reported CAM scores in children with HAs, we added measures of unaided (available for all participants, N = 37) and aided (available for a subgroup, N = 25) hearing thresholds as predictors to our statistical model, but neither factor improved the model fit. We therefore conclude that neither unaided nor aided hearing thresholds have any predictive value for parent-reported CAM scores. Previous studies have reported increased levels of alexithymic traits in deaf and hard-of-hearing adults, but these were measured with self-reported TAS-20 (Peñacoba et al. 2020; Blose & Schenkel 2022). Due to the differing participant populations (children versus adults) and different methodologies (parent-reported CAM versus self-reported TAS-20), it is difficult to make a direct comparison. Furthermore, these studies have not looked into correlations between the degree of hearing loss, or aided thresholds, and the level of alexithymic traits in their samples. All combined, in children with HAs and in our limited sample size, there seems to be no systematic change in parent-reported CAM scores with varying unaided or aided thresholds.

While aided thresholds indicate how well soft sounds can be detected, audibility more specifically refers to how much of the sound energy falls above those thresholds. In this study, it was not possible to verify audibility through measures such as the Speech Intelligibility Index, which have been used in other studies (Kirby et al. 2015; Flaherty et al. 2021). Although previous research has shown that audibility is critical for speech understanding (Scollie 2008; McCreery & Stelmachowicz 2011; Stiles et al. 2012; McCreery et al. 2020), it is possible that basic sound detection increased via amplification may suffice to enhance vocal emotion recognition. Without a formal verification of HA fittings, it is difficult to pinpoint which aspects of amplification and audibility may have an influence on the vocal emotion recognition scores in children with HAs. While all children in the study were fitted with HAs by qualified audiologists following standard clinical protocols and prescriptive targets recommended by fitting software (Scollie et al. 2005; Marriage et al. 2018), research suggests that even when prescriptive targets are followed, actual HA gain can vary significantly, especially for children with moderately severe to profound hearing loss (McCreery et al. 2013; Folkeard et al. 2020; Amri et al. 2022). Future studies may include formal HA fitting verification to assess the impact of actual HA gain on vocal emotion recognition.

### Vocal Emotion Recognition

In this study, with the EmoHI scores for the children with HAs extracted from the previous study, we did not find a significant effect of age. This deviates from the report by Rachman et al. (2025), who examined the EmoHI scores of the larger cohort of 55 children with HAs, among which the 37 children with HAs in this study, and compared them with the EmoHI scores of 86 age-matched children with NH. Their results demonstrated that both groups of children showed a developmental effect, with an increase in EmoHI scores, that is, improved vocal emotion recognition, as a function of age. Given the lack of an age effect on EmoHI scores, it is possible that in this specific study sub-sample, parent-reported CAM and psychophysically measured EmoHI scores are not systematically affected by age due to a lack of power or due to the manifestation of alexithymia traits being relatively stable across the age range of these participants. Alternatively, and as mentioned previously, due to the nature of the parent-report questionnaire, it is possible that the CAM scores of children with HAs are already implicitly normalized with respect to the participants’ age. Parents may fill in the questionnaire with an internal reference in mind for what they consider appropriate behavior for their child’s age and hearing status.

The previously reported data on vocal emotion recognition (Rachman et al. 2025) had shown that, at the group level, children with HAs had lower EmoHI scores, that is, more difficulty recognizing vocal emotions, compared with age-matched children with NH. Within the data extracted for the subgroup of children with HAs, this study showed no evidence for an association between CAM and EmoHI scores using a classical correlation test. This suggests that HA use and the effects of hearing loss likely have an acute and direct impact on vocal emotion recognition, rather than long-term impact on higher order processing stages that may affect both vocal emotion recognition and the development of alexithymic traits. To our knowledge, only 2 studies have investigated alexithymia in deaf adults (Peñacoba et al. 2020; 17 to 66 yr) or adult HA users (Goy et al. 2018; 67 to 94 yr), both using the self-report measure TAS-20. Peñacoba et al. (2020) reported higher levels of alexithymic traits in adults with moderate to profound hearing loss compared with NH individuals, but did not find differences in other measures of emotional functioning, such as the ability to understand one’s feelings and the ability to regulate one’s emotional state as measured by the Trait Meta-Mood Scale. One important difference between the population tested by Peñacoba et al. and the population tested in the current study, beside age, is the degree of hearing loss. Peñacoba et al. tested individuals with mostly severe to profound hearing loss (91% of their participants), while the current study included a relatively larger number of children with less severe degrees of hearing loss (59.5% of the participants had moderate to moderately severe hearing loss). While our results did not show any contribution of unaided and aided PTA4 to the variability in the parent-reported alexithymic trait levels in our sample, the degree to which alexithymic traits are present in child and adult populations with various degrees of hearing loss should be further investigated in larger cohorts to be able to draw any conclusions on a potential relation between the level of alexithymic traits and hearing loss. Moreover, all children in the current study used bilateral HAs, whereas only 42.8% of the participants in the study by Peñacoba et al. used HAs and 6.2% used a cochlear implant, such that more than half of their participants did not make use of any hearing device. This difference may have effects on other aspects besides emotional functioning and identifying emotions, such as language skills, which in turn may also lead to more difficulties in describing one’s emotional state.

### Language Abilities and HA Use

A recent meta-analysis, including studies with both children and adults, found a modest association between language deficits and alexithymic traits, as measured mostly through self-reports (28 of 29 included studies used self-reports, 1 study used parent reports; Lee et al. 2022). There is a possibility that the elevated levels of alexithymic traits seen in young children with HAs, as reported by parents, may be driven by such early challenges in language development. Such an association could be further explored in future research by considering early language development over a longer period of time in children with HAs or by taking into account clinical assessment of language skills.

A mediating role in the effect of language skills on emotional processing could be the consistency with which hearing devices are used. A consistent use of hearing devices is crucial to gain sufficient accumulated auditory experience. The cumulative auditory experience model highlights the importance of early auditory exposure and consistent access to qualitatively rich language interactions for the development of communication, language, and executive functioning in children with hearing loss (Moeller & Tomblin 2015; McCreery & Walker 2022). In addition, caregiver use of mental state language during toddler interactions has been linked to children’s ability to understand emotions (Taumoepeau & Ruffman 2006). Children with moderate hearing loss, in particular, may face disadvantages compared to their peers with NH, especially in the development of empathy, placing them at higher risk for social-emotional challenges (Dirks et al. 2017). Vocal emotion recognition depends not only on the ability to access or perceive relevant acoustic cues but also on interpreting these cues and mapping them to the correct emotional categories. This process requires sufficient exposure to emotional expressions and opportunities for incidental learning. Successful identification of an emotion thus reflects a higher order decision-making stage of emotion recognition, following both perception and interpretation of auditory input. This higher order emotion processing stage can be affected by hearing loss both immediately and over time. While the specific influence of early language input on vocal emotion recognition has not yet been directly studied, it is possible that differences in early auditory experience contribute to the variability observed in emotion recognition abilities. Because we did not explicitly ask parents about their child’s HA use in daily life throughout the past couple of years, we cannot rule out potential effects from the amount and consistency of HA use during early childhood. Previous literature does indicate that school-age children tend to use their HAs more consistently than younger children (Persson et al. 2020; Nailand et al. 2022; Walker et al. 2013), which may contribute to the current finding of younger HA users showing increased levels of alexithymic traits, as measured through parent reports, compared with young children with NH. It would therefore be very valuable to take long-term factors, such as HA use and language abilities in early childhood, into account in future studies.

Furthermore, contrary to the current findings, Goy et al. (2018) did find an association between self-reported alexithymic traits and vocal emotion recognition scores in adult HA users. While there are several differences between the study by Goy et al. and the current one, such as the participants’ age range and the use of the self-reported TAS-20 (Goy et al. 2018) versus the parent-reported CAM (current study), another difference seems to be the relatively higher prevalence of alexithymic traits in the older HA users. Goy et al. reported a mean and SD of 52.8 and 6.2, respectively (range = 39 to 63), out of the total possible range of 20 to 100, where scores of 52 or greater indicate possible alexithymia (TAS-20; Bagby et al. 1994).

In this study, alexithymia was explored in school-age children with HAs from a young age, 5 yr, an important period for language and cognitive development. Such an exploration was possible due to the use of the CAM, which was designed for assessment in young children via parent reports. On the other hand, we should be cautious about the methodology used. The CAM was originally developed as an instrument for children with a history of trauma (Way et al. 2010), and later used in populations with clinical disorders related to social functioning, such as autism spectrum disorder. Moraitopoulou et al. (2024) used the CAM in relatively large populations of adolescents (10 to 16 yr) with and without autistic traits (N = 75 and N = 109, respectively) and did not find any correlation between parent-reported CAM scores and facial emotion recognition accuracy scores. Their findings deviate from often reported associations between emotion recognition from facial expressions and levels of alexithymic traits in adult populations (for a review, see Grynberg et al. 2012). Moraitopoulou et al. discuss several potential explanations for this discrepancy, such as the idea that difficulties related to alexithymia may only emerge during the later teenage years and early adulthood. Furthermore, they point to the possibility that while parent-report measures are appropriate for the assessment of younger children, they may miss out on more internal manifestations of alexithymia. Rieffe et al. (2006) had adapted TAS-20 to use with children, also via self-report, namely, Alexithymia Questionnaire for Children (Rieffe et al. 2006). This questionnaire was validated only for children 9.5 yr and older, and therefore not usable with young populations like in our study. In our study, we saw a group effect only in children up to 8.8 yr, such that the children’s version of TAS-20 likely would not have been able to capture this effect. Our finding that there was no difference in the level of alexithymic traits between children with HAs and children with NH is not in line with previous reports with adult populations with hearing loss using TAS-20. This may indicate that potential hearing loss and alexithymia interactions differ between children and adults with hearing loss, or that the differing methodologies, such as self or parental reporting, capture different patterns. For a better understanding of the different aspects of alexithymia that are measured with TAS-20 and CAM, follow-up research could make use of both questionnaires in a sample of teenagers.

### Summary

In summary, based on group comparisons, our findings suggest that the children with HAs in this study did not exhibit increased levels of alexithymic traits as measured through parental reports using the CAM. However, once analyzed for age, the 2 groups showed different developmental trajectories, which seemed to be mostly in ages younger than 8 yr, and with no developmental effect in children with HAs. This may indicate some difference in the level of parent-reported alexithymic traits in younger children with NH and with HAs, where children with HAs perhaps show a reduction in the level of alexithymic traits with time, to the degree of what is expected for their age. On the other hand, the absence of an effect of aided and unaided thresholds on parental reports of alexithymic traits and the overlap of CAM scores for both groups in this study with the CAM scores reported in literature for neurotypical children support the idea that perhaps hearing loss alone is not directly associated with alexithymic traits in children. Finally, the absence of a correlation between CAM and EmoHI scores suggests that difficulties in vocal emotion recognition may be restricted to the auditory perception of emotion-related voice cues and are less likely to be due to long-term effects leading to difficulty recognizing and describing feelings as measured by parental reports of the level of alexithymic traits using the CAM.

## ACKNOWLEDGMENTS

The authors are grateful to all children, parents, students, and colleagues who participated in this study, the Turkish Ministry of National Education (T.C. Milli Eğitim Bakanliği) for general support, and the Hacettepe University audiology clinic, state schools, hearing aid shops, and rehabilitation centers for their support with the recruitment of participants. The authors also like to thank Paolo Toffanin, Leanne Nagels, Iris van Bommel, Evelien Birza, Jacqueline Libert, Jemima Phillpot, Marta Matos Lopes, and Jop Luberti (illustrations) for their contribution to the development of the child-friendly game-like interface, the authors’ children and children’s friends for their help with piloting and improving the initial versions of the test, Jennifer Breetveld, Wijnna Beuving, Gesina Posthumus-Ottema, Esther Steenbergen for research support, Christina Elsenga for help in data sharing, Monita Chatterjee for feedback on the manuscript, and Soner Türüdü for help identifying mistakes and modifying the Turkish translation of the CAM.

## Footnotes

* Upon completion of the study, in the original version of CAM translation, a number of typos were identified. These are remedied and corrected in a new version of the CAM translation. Both versions are available upon request.
